# Attentional and lexical factors underlying word-centred neglect dyslexia errors in healthy readers

**DOI:** 10.3758/s13414-023-02753-x

**Published:** 2023-07-06

**Authors:** James Gurd, Nele Demeyere, Margaret Jane Moore

**Affiliations:** 1 Department of Experimental Psychology, University of Oxford, Oxford, UK; 2 Queensland Brain Institute, University of Queensland, Brisbane, QLD Australia

**Keywords:** Attention, Reading, Neuropsychology

## Abstract

Word-centred neglect dyslexia is most commonly conceptualised as a deficit caused by attentional biases within spatially coded internal representations of words. However, recent research has suggested that at least some cases of word-centred neglect dyslexia are unrelated to visuospatial neglect and may instead be modulated by self-inhibition and lexical factors. Here, we set out to provide novel insight into potential underlying mechanisms modulating the occurrence of word-centred lateralised reading errors in healthy participants. A sample of 47 healthy readers completed a novel attentional cueing paradigm in which they sequentially identified lateral cues and read presented words under limited exposure conditions. Reading responses were analysed to determine whether word-centred neglect dyslexia could be simulated in healthy readers, to compare the strengths of induced biases, and to identify systematic differences in lexical characteristics between target words and neglect dyslexia reading errors. Healthy participants produced frequent lateralised reading errors in both horizontal and vertical reading stimuli with > 50% of errors classed as neglect dyslexic. Cues appended to word beginnings elicited significantly more reading errors than cues at word ends, illustrating the interaction between existing reading spatial attentional biases and cue-induced biases. Neglect dyslexia reading errors were found to contain significantly more letters per word and had higher concreteness ratings than target words. These findings demonstrate that word-centred neglect dyslexia can be simulated using attentional cues in healthy readers. These results provide important insight into the mechanisms underlying word-centred neglect dyslexia and further fundamental understanding of this syndrome.

**Supplementary information:**

The online version contains supplementary material available at 10.3758/s13414-023-02753-x.

## Introduction

The term *neglect dyslexia* refers to a collection of acquired dyslexias that are characterised by consistently lateralised reading errors. Neglect dyslexia is behaviourally similar to, and often comorbid with, visuospatial neglect, in which a patient fails to respond to stimuli in contralesional space (Vallar et al., 2010). Whilst there have been many detailed case reports of patients with neglect dyslexia (e.g., Ellis et al., 1987; Friedmann & Nachman-Katz, 2004; Riddoch, 1990) and a growing number of systematic reviews and larger cohort analyses (e.g., Beschin et al., 2014; Haywood & Coltheart, 2000; Moore et al., 2020; Moore & Demeyere, 2017; Vallar et al., 2010), the specific cognitive mechanisms underlying neglect dyslexia are not well understood.

**Fig. 1:**
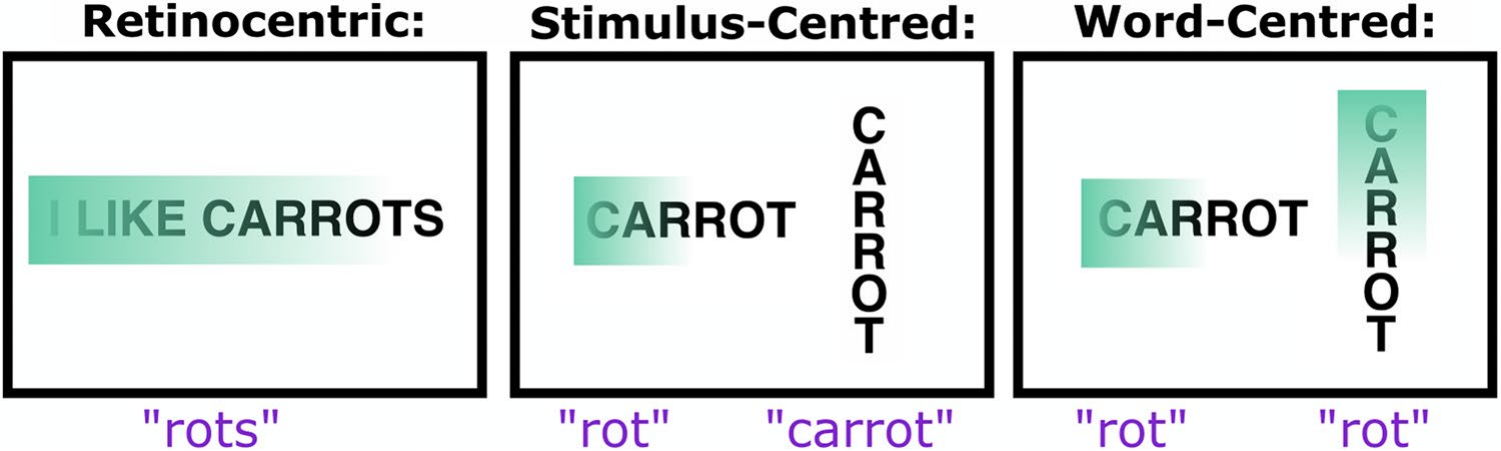
A visualisation of the three levels of neglect dyslexia impairment as classified by Hillis and Caramazza (1995). This illustration exemplifies left-lateralised neglect dyslexia deficit (e.g., Hillis & Caramazza, 1995), but cases of right-lateralised neglect dyslexia have also been documented (e.g., Moore & Demeyere, 2020)

Neglect dyslexia is most commonly conceptualised in accordance with a three-level model proposed by Hillis and Caramazza (1995). This model categorises cases of neglect dyslexia within three distinct reference frames of impairment: retinocentric, stimulus-centred, and word-centred (Fig. 1). In retinocentric neglect dyslexia, patients make reading errors dependent on the egocentric position of the word stimulus. This impairment is exemplified by patient JOD, who was found to make full word omission errors within the left but not the right visual field (Hillis & Caramazza, 1995). Retinocentric neglect dyslexia is generally content-unspecific and is often associated with severe egocentric visuospatial neglect (Beschin et al., 2014; Moore et al., 2020).

Conversely, stimulus-centred neglect dyslexia involves reading errors characterised by consistently lateralised letter omissions, additions, and substitutions when reading individual words (Haywood & Coltheart, 2000; Hillis & Caramazza, 1995). Critically, stimulus-centred neglect dyslexia occurs regardless of each stimulus’ location in egocentric space (Hillis & Caramazza, 1995). For example, patient VB committed left-lateralised letter omissions when reading horizontal words presented in the right and left visual fields (Ellis et al., 1987). However, VB exhibited no reading impairment when reading vertically presented words (Ellis et al., 1987). This error pattern is characteristic of stimulus-centred neglect dyslexia, and is thought to occur due to spatial-attentional impairment within an allocentric frame (Hillis & Caramazza, 1995).

Finally, word-centred neglect dyslexia is defined by the occurrence of letter reading errors relative to spatially coded internal representations of words (Hillis & Caramazza, 1995). Word-centred neglect dyslexia errors are unaffected by topographical stimulus-centred manipulations, and always impact the same region of words regardless of where those letters are presented in space (e.g., in vertical presentations; Caramazza & Hillis, 1990). Patient NG, for example, made reading errors in the terminal portions of horizontal, vertical and reflected word stimuli, even though in a reflected condition the terminal portion is on the opposite side of egocentric space (Caramazza & Hillis, 1990). Although Caramazza and Hillis (1990) initially attributed this impairment to an attentional deficit within a word-centred coordinate system for grapheme information, recent research has suggested that at least some cases of word-centred neglect dyslexia are unrelated to the broader neglect syndrome.

Several previous case studies have documented neglect dyslexia impairment occurring in the absence of similarly lateralised visuospatial neglect. For example, Friedmann and Nachman-Katz (2004) identified a left neglect dyslexia in a 10-year-old boy with no history of neurological disorder and no evidence of visuospatial neglect impairment. Similarly, Schubert and McCloskey (2013) reported a case of right-word centred neglect dyslexia occurring within a patient with no neglect impairment. Moore and Demeyere (2019) describe a single case, AB, who demonstrated left word-centred neglect dyslexia in the absence of egocentric or allocentric visuospatial neglect. Patients CD (Moore & Demeyere, 2020) and EF (Moore & Demeyere, 2023) were found to exhibit clear left domain-general neglect in conjunction with right-lateralised word-centred neglect dyslexia. This dissociation in neglect lateralisation is not predicted by Hillis and Caramazza's (1995) three-tier model as this theory asserts that the same spatial-attentional deficit underlies neglect deficits in lexical and non-lexical stimuli. Further, these patients’ reading errors were not modulated by attentional factors (exposure time and letter spacing), known to modulate symptoms of visuospatial neglect (Ellis et al., 1987; Husain & Kennard, 1997; Riddoch, 1990). These occurrence and lateralisation dissociations between neglect and word-centred neglect dyslexia, not predicted by Hillis and Caramazza’s (1995) three-tier model, indicate that some cases of word-centred neglect dyslexia may involve distinct underlying impairments. These recent findings suggest that neglect impairment alone is insufficient for explaining all cases of neglect dyslexia, and that additional research is needed to clarify the mechanism underlying word-centred neglect dyslexia.

Previous case studies have provided preliminary evidence that some cases of neglect dyslexia that occur in the absence of neglect may involve a deficit of self-inhibition (Moore & Demeyere, 2020, 2023). For example, patient EF exhibited right word-centred neglect dyslexia (in line with the criteria outlined by Hillis & Caramazza, 1995) in conjunction with left lateralised egocentric and allocentric neglect (Moore & Demeyere, 2023). EF demonstrated an intact ability to identify all letters in presented words, but reliably committed neglect dyslexia errors when subsequently reading the same words as a whole. These neglect dyslexia reading errors were characterised by misreading less familiar target words as more familiar responses. The severity of this deficit was not found to be modulated by factors established to exacerbate the severity of visuospatial neglect (e.g., exposure time, spacing, location manipulations) (Moore & Demeyere, 2023). These findings strongly suggest that EF’s reading impairment cannot be explained as a side effect of visuospatial neglect, but instead may be related to a distinct cognitive deficit. Importantly, this error pattern cannot be accounted for by merely ‘filling in’ missing information as EF exhibited a spared ability to identify all letters in presented words and exhibited no impairment when reading non-lexical stimuli (e.g., letter strings).

Instead, EF’s cognitive assessment data provide preliminary evidence that some cases of word-centred neglect dyslexia may be related to impairments in cognitive inhibition. EF’s word-centred neglect dyslexia co-occurred with a marked deficit of cognitive inhibition as reported by a battery of standard cognitive assessments. Specifically, EF exhibited pronounced impairment within the Hayling Sentence Completion task, which assesses the ability to inhibit pre-potent responses associated with verbally presented sentence fragments (Bielak et al., 2006; Moore & Demeyere, 2023). EF also exhibited an inability to inhibit pre-potent responses within Stroop tasks, a marked impairment in rule switching components of verbal/design fluency and trail making tasks (Delis et al., 2004), and an inability to shift away from learned rules in standard rule-switching tasks (Humphreys et al., 2012). Based on these findings, Moore and Demeyere (2023) suggest that EF’s word-centred neglect dyslexia may involve inhibitory deficits rather than the neglect impairment.

Cognitive inhibition refers to the ability to override or halt dominant responses (Chiappe et al., 2000; Diamond, 2013; Guarino et al., 2020). Previous work has demonstrated that cognitive inhibition plays an important role in facilitating normal reading by reducing interference from irrelevant information and by preventing dominant responses from seizing control of action and thought (Chiappe et al., 2000; Diamond, 2013; Guarino et al., 2020). Moore and Demeyere (2023) posit that these processes, when disrupted, could plausibly account for the pattern of impairment seen in word-centred neglect dyslexia. Healthy readers exhibit strong processing advantages for initial letters over later letters in presented words (Scaltritti et al., 2018; Scaltritti & Balota, 2013). Due this processing imbalance, cognitive inhibition is needed to suppress dominant responses associated with the first-processed letters until additional information from subsequent letters is adequately considered (Diamond, 2013). For example, when presented with a comparatively unfamiliar stimulus (e.g., COVEN), EF was hypothesised to begin encoding graphemes from left-to-right then erroneously report the first (more accessible/familiar) word that matched these first-processed letters (e.g. linking ‘C-O-V-’ to COVER) rather than considering all presented graphemes. Therefore, word-centred neglect dyslexia may arise, when information from first-processed letters is employed while information from latter letters is ignored, regardless of stimulus orientation (Moore & Demeyere, 2023).

Although past case study work suggests a potential inhibitory mechanism, additional research is needed before this conceptualisation can be confidently supported or refuted. Word-centred neglect dyslexia is a comparatively rare and heterogenous impairment (Moore & Demeyere, 2017; Vallar et al., 2010). These factors make it extremely challenging to conduct group studies of this deficit, which may preclude drawing generalisable conclusions about the syndrome as a whole. Considering data from healthy individuals offers a potential avenue to further understanding of word-centred neglect dyslexia. Mainly, identifying parameters which create neglect dyslexia-like errors in healthy adults can help provide insight into the specific mechanisms that are disrupted in patients with word-centred neglect dyslexia.

This study employs an attentional cueing paradigm to investigate whether word-centred neglect dyslexia errors can be simulated in healthy adults. Reading single words inherently involves some degree of spatial attentional bias, regardless of spatial attentional deficits (Scaltritti & Balota, 2013). When reading English words, attention is biased towards the initial (usually left-lateralised) word letters (Gabrieli & Norton, 2012; Henderson, 1992; Scaltritti & Balota, 2013). This bias occurs despite the fact that individual letters within words are generally processed in parallel rather than sequentially (Jackson & Coltheart, 2013). If word-centred neglect dyslexia involves deficits unrelated to spatial attention, this bias can be expected to play a key role in predicting errors as patients would be expected to respond based on the letters processed first, regardless of word orientation (Moore & Demeyere, 2020, 2023). Attentional cueing can also provide insight into the role that this bias may play in producing word-centred neglect dyslexia errors. If inhibition plays a key role in modulating the occurrence of errors, errors can be expected to occur most frequently when spatial bias created by attentional cueing is congruent with spatial bias towards the initial letters of word stimuli (Scaltritti & Balota, 2013). Conversely, word-centred neglect dyslexia errors can be expected to be less frequent when these two sources of spatial-attentional bias are incongruent. For example, by this account, more terminal omission errors would be expected when cues are presented near initial words whilst more initial omissions would be expected when cues are presented near terminal letters.

Finally, systematic differences in lexical characteristics between target words and neglect dyslexia errors may help to provide insight into the mechanisms behind error responses (Moore & Demeyere, 2020). If word-centred neglect dyslexia represents an inhibitory deficit, neglect dyslexia errors could be characterised by misreading less familiar words as more familiar words and/or less concrete words as more concrete words. This is because these stimuli may be expected to have more probable incongruent, pre-potent responses compared to more common words. In other words, less familiar words often have more familiar words that are visually similar that must be inhibited for a correct response to be made (Davies, 2009). There are several different lexical characteristics that may help provide proxy measures of familiarity. Spoken and written word frequency metrics can help estimate how regularly participants are externally exposed to words, whilst measures including concreteness, semantic density, age of acquisition, and number of orthographic/phonological neighbours can help estimate how easily accessible words are to participants (Balota et al., 2007; Moore & Demeyere, 2020; Riddoch et al., 1990). Clarifying how these different lexical factors modulate neglect dyslexia reading patterns may also clarify the mechanisms behind the syndrome.

The purpose of this study was to determine whether word-centred neglect dyslexia can be simulated in participants by manipulating attentional biases: The study investigated the complementary roles of attentional cueing, inherent spatial-attentional reading biases, and lexical factors. If the deficit is able to be successfully simulated, examining the lexical and spatial parameters that modulate the occurrence of word-centred neglect-dyslexia errors can provide novel insight into the precise mechanisms underlying this impairment. This study aimed to expand on the design of previous studies that have employed attentional cueing paradigms in healthy individuals to further fundamental insight into information processing biases in clinical cases of neglect dyslexia (Behrmann et al., 1991). Past case study work has demonstrated that lateralised attentional cues modulate the severity of clinical cases of neglect dyslexia (Behrmann et al., 1990; Cubelli & Beschin, 2005; Riddoch et al., 1990), suggesting that manipulating these parameters may provide further insight into the specific cognitive mechanisms driving this disorder. Overall, this study aimed to further the fundamental understanding of neglect dyslexia, which is a necessary precursor of effective rehabilitation for patients impacted by neglect dyslexia reading deficits.

## Methods

### Participants

A sample of healthy, normal readers were recruited from within the Oxford Research Participation Scheme. This protocol was approved by the University of Oxford Medical Sciences Interdivisional Research Ethics Committee (R70527/RE001). Participants were required to be over the age of 18 years, willing and able to give informed consent, have sufficient understanding of English, have normal or corrected-to-normal vision, and not to have identified or diagnosed reading or hearing difficulties. Of the 50 participants who completed the study, three were excluded due to incomplete audio data due to technical difficulties. The data for analysis therefore came from 47 adults (23 male, 46 right-handed), with a mean age of 24.53 years (SD *=* 10.97 years, range = 18–56 years). This sample size was arbitrarily determined by roughly tripling the sample size of previous investigations aiming to employ data from healthy individuals to explore the mechanisms underlying neglect dyslexia (Behrmann et al., 1991). No a priori power analysis was conducted for this study due to the lack of a reasonable expected effect size benchmark.

### Methods and materials

**Fig. 2:**
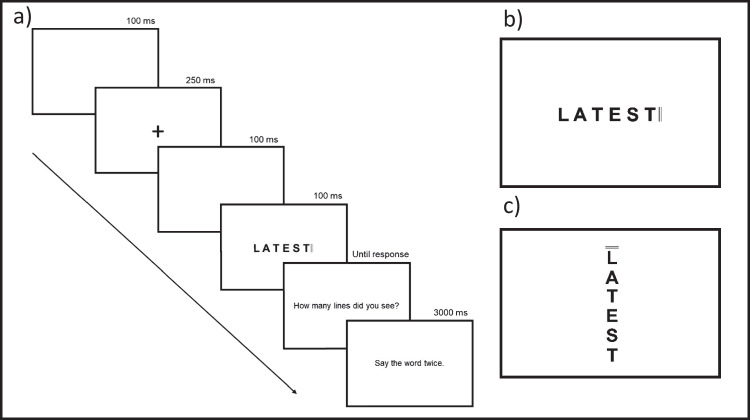
A visualisation of the experimental paradigm employed in this study. Panel **a** demonstrates the time course of each individual trial, panel **b** provides an example of a horizontal stimulus with a three-lined terminal cue, and panel **c** presents an example vertical stimulus with a two-lined initial cue. All stimuli are matched for font, size and luminosity

This investigation employed a novel attentional cueing paradigm in which participants were required to identify lateral cues and read aloud simultaneously presented words under reduced exposure conditions. In each trial, a single word was presented in either horizontal or vertical orientation accompanied by one of two potential cues, appended to the beginning or end of the presented word stimuli (Fig. 2). Word stimuli were printed in 60-pt Arial capital letters. As this experiment was conducted online, screen resolution varied across participants, but stimulus visual angle was standardised relative to each participant’s screen and viewing distance using Gorilla Experiment Builder’s built in calibration functions. In this standard calibration procedure, participants are instructed to sit approximately 60 cm directly in front of their computer screen and to scale an onscreen image of a bank card to match the exact size of their own bank card. Built-in Gorilla function employs this information to ensure that the visual angle of all presented stimuli (23.2°) was matched across all monitors used in data collection. Attentional cues were either double or triple lines appended to either the beginning or end of word stimuli (see Fig. 2). Each completed stimulus was size standardised to 14 cm in length. These stimuli remained on screen for 100 ms, and participants were immediately asked to report which cue had been presented, and subsequently report the presented stimulus words. Each participant completed 720 trials separated into four blocks.

Each block contained stimuli in one orientation (either horizontal or vertical) with cues at one of the two potential locations (either terminal or initial) yielding four distinct block conditions. This approach was adopted as it was expected to more consistently bias attention to one side instead of forcing participants to switch the lateralisation of their focus on each trial. The order of the blocks was randomised between participants using a Latin square design (Winer, 1962). Each block contained the same 180 words (all were six-letter words; see Online Supplementary Material (OSM) Table 1). These words had a normative mean log frequency of 7.98 (SD = 2.2, range = 1.4–12.9), a mean subtitle word frequency of 32.8 (SD = 131.6, range = 0.04–1500.2), an average of 2.7 (SD = 3.07, range = 0–14.0) orthographic neighbours, an average of 7.98 (SD = 2.21, range = 1.39–12.9) phonological neighbours, a mean concreteness rating of 4.9 (SD = 5.9, range = 0–28), a mean semantic density rating of 3.66 (SD = 1.1, range = 1.4–5.0), and an average age of acquisition score of 0.52 (SD = 0.15, range = 0.16–0.69).

Participants were not informed that words would repeat in each block. These words were selected such that a new, real word could be read by omitting one, two or three letters from the end of the word, and a different new word could be read by omitting one, two or three letters from the beginning (e.g., LATEST could be read as either LATE or TEST). Efforts were also made to minimise homophonous neglect dyslexia-like errors (e.g., PLEASE might be misread as ‘PLEAS’, but this is not audibly different).

Each individual trial consisted of presentation of a fixation cross for 250 ms, followed by the presentation of a stimulus for 100 ms. Participants then had an unlimited amount of time to respond whether the cue contained two or three lines by pressing ‘2’ or ‘3’ on their keyboard, followed by 3 s to say the word aloud twice. They were asked to read words twice to ensure audio capture of their full response. Audio recordings of each response were made using each participant’s built-in computer microphone. Audio recording commenced alongside onset of the reading response prompt (see Fig. 2) and continued until manually ended by the participant (by clicking to begin the next trial). As the reading prompt was displayed after the delay caused by the cue response prompt, these recordings were not of sufficient precision to reliably calculate reading latencies but were of sufficient quality to facilitate response accuracy coding.

The experiment was created and run online using the Gorilla Experiment Builder (www.gorilla.sc). Participants used their own devices for the study, and they calibrated their screens using Gorilla Experimental Builder built-in functions to ensure that stimuli appeared at the right size (14 cm, visual angle = 23.2°). Each block was preceded by five practice trials and each block was separated by a break period of up to 5 min. The entire experimental paradigm took around 60 min to complete. After completion, participants were debriefed about the aims of the study and either received payment or course credits. No part of this protocol was preregistered.

### Data coding and categorisation

First, all audio data was transcribed by an investigator (JG) who was blinded to the target word, condition, cue and participant. In cases where participant’s first response differed from their second, only their first response was recorded. Where possible, audio responses were interpreted as real words (e.g., as ‘FOREMAN’ rather than ‘FORMAN’ or ‘FOURMAN’). Where target words permitted homophonous incorrect responses, audio was interpreted to match the target rather than an error (e.g., as ‘HEARTS’ not ‘HARTS’). This approach was adopted as homophonous responses are indistinguishable based on auditory data alone. Unintelligible responses were removed from all subsequent analyses.

Next, all transcribed responses were compared to the target words to binarise response accuracy, and error responses were categorised. Reading errors were classified as lateralised where the reading error was restricted to one half of the target, irrespective of the letters involved in the error. These lateralised errors were defined with respect to terminal/initial letters of words rather than to the spatial orientation of the presented stimulus. This category included addition, substitution and omission neglect dyslexia errors as defined within previous literature (Moore & Demeyere, 2017; Vallar et al., 2010). Errors were categorised as addition when responses added additional terminal or initial letters (e.g., FORMAT read as ‘FORMATTED’), as substitution when terminal/initial letters were replaced by incongruent letters (e.g., FORMAT read as ‘FORMED’), and omission when terminal/initial letters were omitted from responses (e.g., FORMAT read as ‘FORM’) (Vallar et al., 2010). All lateralised errors were coded as to whether the error was in the *initial* or *terminal* portion. Any other error was classed as *non-lateralised*.

### Statistical Analyses

First, the coded reading data was cleaned to ensure quality. Data from individual participants were excluded from the study if their accuracy when reporting cue identities was less than 60%, or if they were missing more than 50% of their audio data. Next, general descriptive statistics were calculated to summarise overall performance on the experimental paradigm. Chi-squared tests were conducted to compare accuracy statistics across the two orientation and two cueing conditions. Participant accuracy was calculated for both reading and cue responses and the frequency of each categorised error type was reported.

Next, the spatial lateralisation of reading errors was analysed in order to determine whether the experimental paradigm was able to simulate word-centred neglect dyslexia errors on both a group and a participant level. There are no established impairment thresholds for determining whether or not a patient exhibits significant neglect dyslexia impairment (Moore & Demeyere, 2017; Vallar et al., 2010). In previous word-centred neglect dyslexia case studies, neglect dyslexia reading errors have represented between 43.9% and 95.8% of total reading errors across a range of reading tests (Moore & Demeyere, 2019, 2020, 2023). For this reason, word-centred neglect dyslexia was considered to be successfully simulated if neglect dyslexia errors composed at least 45% of errors within both the vertical and the horizontal reading conditions. The spatial lateralisation of reading errors was analysed to determine whether neglect dyslexia could be simulated within both horizontal and vertical stimuli and in both the left and the right lateralization. In each case, error proportions were analysed using a generalised estimating equation (GEE) as a non-parametric alternative to ANOVA statistical tests. GEE models enable power extraction of population-average effects, without making assumptions about data distributions (Ghisletta & Spini, 2004). This approach is appropriate for the reading data collected in this investigation as group-level accuracy data may be non-parametric. In these models, errors are conceptualised as either cue-congruent (e.g., an initial error in a terminal cueing condition) or cue incongruent (e.g., a terminal error in a terminal cueing condition).

Three GEE models were created. In each case, the outcome variable was the number of initial errors as a proportion of lateralised errors. Model 1 addressed only trials with words presented horizontally; the predictor variables were cue lateralisation, the proportion of non-lateralised errors, and cue accuracy (the average cue accuracy for an item across participants). Model 2 addressed only trials with words presented vertically; the predictor variables were cue lateralisation, the proportion of non-lateralised errors, and cue accuracy. Model 3 considered all trials and considered word orientation, cue lateralisation, the interaction between orientation and the proportion of non-lateralised errors, and cue accuracy as predictor variables.

Finally, a series of analyses were conducted to identify systematic lexical characteristic differences between target and response words in cases where participants committed lateralised reading errors. All employed lexical and semantic characteristics were obtained via the English Lexicon Project database (Balota et al., 2007). This analysis included the variables: log word frequency, subtitle word frequency, word concreteness, orthographic neighbourhood, and phonological neighbourhood, as these variables have been analysed in previous studies of word-centred neglect dyslexia (Moore & Demeyere, 2020, 2023) . Log word frequency was calculated from the values assigned to frequency in analogue to language model of lexical semantics (see Burgess, 1998), where larger values indicate more common words (lexicon mean = 4.49, range = 0–17, SD = 2.89). Subtitle word frequency estimates the frequency of words in spoken language by scoring each word’s frequency per million words used in television programmes and films (New et al., 2007) (lexicon mean = 25.23, range = 0–41,857, SD = 467). Word concreteness describes the extent to which the semantic denotation of a word refers to a perceptible entity (Brysbaert et al., 2014). The concreteness scores are based on the ratings collected by Brysbaert and colleagues (lexicon mean = 3.11, range = 1.04–5, SD = 1.03). Importantly, concreteness can also act as an effective proxy measurement of ‘familiarity’ as this metric helps capture how easily words can be accessed (Brysbaert et al., 2014; Miller & Roodenrys, 2009). Orthographic neighbourhood refers to the number of words that are the same length as a target and differ by one letter, for example, FAST and TACT are orthographic neighbours of fact (Huntsman & Lima, 2002) (lexicon mean = 1.58, range = 0–34, SD = 3.34). Phonological neighbourhood refers to the number of words that differ from the target by one phoneme (Luce & Pisoni, 1998) (lexicon mean = 3.43, range = 0–67, SD = 7.75).

First, a single-sample, two-tailed t-test was conducted to compare the length of lateralised error responses to the length of the target stimuli. Next, an additional logistic regression was conducted to determine if any of the considered lexical characteristics were systematically different between target and lateralised error responses. This model considered stimulus category (target/response) as the outcome variable and employed log word frequency, subtitle word frequency, orthographic neighbourhood, phonological neighbourhood, and concreteness as covariates. Given that this regression involved five individual significance tests, a Bonferroni-corrected alpha level of 0.01 was used.

#### Open science practices statement

All experimental materials, data, and analysis code for this project are available on the Open Science Framework (www.osf.io/nkzb8) (Foster & Deardorff, 2017). The reported experiments were not preregistered.

## Results

### Performance descriptives

All participants obtained cue reporting accuracies of over 65%, and therefore none were excluded on the grounds of cue accuracy. Sex, age, and handedness were not found to significantly predict accuracy within cue-recognition or word-reading. Overall, participants exhibited a mean cue accuracy of 83.86% (SD = 6.49%, range = 69.2–94.6%). Cue accuracy was greater for horizontal words (M = 89.80%, SD = 5.20%, range = 75.9–96.2%) than for vertical words (M = 77.73%, SD = 9.49%, range = 55.9–94.4%) (χ^2^ (1) = 5.36, p = .206). Cue accuracy was not significantly different for initial cues (M = 86.81%, SD = 6.83%) versus terminal cues (M = 81.11%, SD = 8.09%) (χ^2^ (1) = 1.21, p = .271).

Within correct cue response trials, participants had an average reading response accuracy of 86.90% (SD = 8.95%, range = 65.06–99.65%). Average reading response accuracy was greater for horizontal words (M = 93.57%, SD = 4.91%, range = 81.67–91.32%) than for vertical words (M = 79.69%, SD = 14.72%, range = 42.55–98.23%) (χ^2^(1) = 8.31, p = 0.004). When data from horizontal and vertical conditions were combined, reading accuracy was not found to be significantly different between initial cue words (M = 86.74% (SD = 9.83%, range = 55.77–99.07%) and terminal cue words (M = 87.61%, SD = 9.51%, range = 55.67–99.65%) (χ^2^ (1) = 0.03, p = 0.854). Within the horizontal reading condition, a significantly higher proportion of errors were cue-congruent neglect dyslexia reading errors in initial cue stimuli compared to terminal cue stimuli (155/676 vs. 145/982, respectively, χ^2^(2) = 18.0 p < 0.001). A similar relationship was present within vertical stimuli, with a significantly higher proportion of errors being cue-congruent in initial cueing conditions compared to terminal cuing conditions (651/1415 vs. 265/2041, respectively, χ^2^(2) = 467.8, p < 0.001).

As each block consisted of the same 180 words, a one-way ANOVA analysis was run to determine whether reading performance accuracy was different across blocks (indicating potential learning effects). No significant differences were present in performance when blocks were coded according to the order they were completed in (F(3,184) = 0.729, p = 0.536).

### Simulating neglect dyslexia

**Fig. 3:**
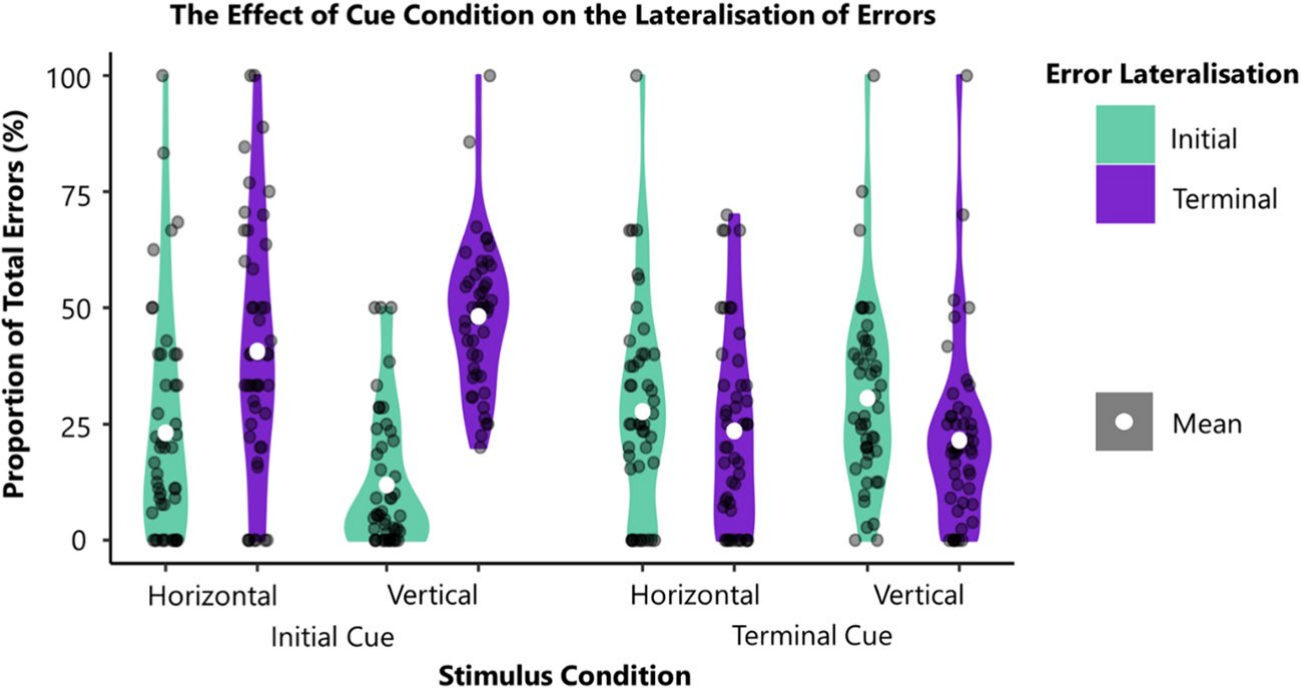
A visualisation of the relationship between the proportion of terminal versus initial errors across different conditions and cue lateralisations. Participant proportions are represented by black dots whilst the group means are marked in white. The distribution of data is represented by the coloured, asymmetrical blobs. Critically, cue-congruent reading errors were more common than cue-incongruent errors across all stimuli types and conditions

In horizontal trials participants committed a total of 2,550 (15.6%) reading errors with 55.86% classed as lateralised reading errors within trials with correct cue responses. The vast majority of reading errors involved misreading the stimulus as a real word, with only 5.56% of errors being non-words. Of the lateralised reading errors, 58.10% were substitution, 10.26% were omission, 6.73% were addition, 6.81% were combined substitution/omission errors (e.g., KEYPAD read as KEYS), and 18.10% were combined substitution/addition errors (e.g., OFFICE read as OFFERING). Across all participants, 56.53% (SD = 34.77%) of lateralised errors were cue-congruent and 38.15% (SD = 33.32%) were cue-incongruent (χ2 (1) = 20.44, p < .00001) (Fig. 3). GEE model 1 demonstrated that, within horizontal conditions cue lateralisation was a significant predictor of error lateralisation (p < 0.01, 95% CI: 1.25– 3.56) (Table 1). Terminal cues were found to be 211% more likely than initial cues to result in initial errors, indicating that cue-congruent errors were more frequent than cue-incongruent errors within horizontal stimuli. Neither the proportion of non-lateralised errors nor cue accuracy had a significant effect on the proportion of initial errors (Table 1).

**Table 1:** Generalised estimating equation (GEE) modelling results and summary statistics for each of the three analysed GEE models. Each analysis considers the number of initial errors as a proportion of lateralised errors as the outcome with the predictor variables listed in each row. Model 1 analyses these effects within horizontal stimuli, Model 2 within vertical stimuli, and Model 3 considers all collected data as a whole. These results indicate that error lateralisation is significantly predicted by cue lateralisation, with cue congruent errors being significantly more likely than cue incongruent errors. Additionally, cue-congruent errors were more common within vertical stimuli than in horizontal stimuli and initial errors were slightly more common in horizontal orientations, regardless of cue location

Model 1: Horizontal stimuli	Beta	SE	p	Odds Ratio	95% CI
(Intercept)	-0.56	0.22	0.01		
Cue Lateralisation	0.74	0.27	< 0.01	2.11	1.25 - 3.56
Non-Lateralised Errors	0.94	0.76	0.21	2.57	0.58 - 11.35
Cue Accuracy	1.74	2.37	0.46	5.72	0.06 - 592.3
Model 2: Vertical stimuli	Beta	SE	p	Odds Ratio	95% CI
(Intercept)	-2.11	0.22	< 0.001		
Cue Lateralisation	2.5	0.28	<0.001	12.18	6.99 - 21.2
Non-Lateralised Errors	-1.29	0.8	0.11	0.28	0.06 - 1.32
Cue Accuracy	1.76	1.38	0.2	5.8	0.38 - 87.5
Model 3: All stimuli	Beta	SE	p	Odds Ratio	95% CI
(Intercept)	-0.78	*0.23*	< .001		
Orientation	-1.2	*0.24*	< .001	0.3	0.19 - 0.48
Cue Lateralisation	0.92	*0.25*	< .001	2.52	1.55 - 4.10
Orientation*Cue Lat	1.48	*0.3*	< .001	4.4	2.46 - 7.86
Non-lateralised Errors	-0.26	*0.6*	0.67	0.77	0.24 - 2.50
Cue Accuracy	1.5	*1.25*	0.23	4.48	0.38 - 52.38

In vertical trials, participants read 5,846 (37.6%) presented words incorrectly, with 55.21% of errors meeting criteria for lateralised reading errors within correct cue response trials. Across all participants, 70.01% (SD = 26.68%) of errors were cue-congruent and 28.93% (SD = 25.84%) were cue-incongruent (χ^2^ (1) = 323.63, p < .00001). GEE Model 2 (Table 1) revealed that cue lateralisation was also a significant predictor of error lateralisation within vertical stimulus trials. In vertical stimuli, terminal cues were 1218% more likely than initial cues to result in initial errors. Neither proportion of non-lateralised errors nor cue accuracy had a significant effect on the proportion of initial errors committed (Table 1).

Finally, GEE model 3 (Table 1) revealed that, across all trials, cue lateralisation remained a significant predictor of error lateralisation. Terminal cues were 252% more likely than initial cues to result in initial errors, indicating that cue-congruent errors were more likely that cue-incongruent errors. Orientation also significantly impacted the probability of initial errors, with 30% more initial errors occurring within horizontal than vertical stimuli (Table 1). There was a significant interaction between word orientation and cue lateralisation, with vertical stimuli with terminal cues being 440% more likely to elicit cue-congruent (i.e., initial) errors than horizontal stimuli with terminal cues. Similar to the individual analyses, the proportion of non-lateralised errors and cue accuracy did not have a significant effect on the proportion of initial errors (Table 1). See Fig. 3 for a visualisation of these data.

### Lexical analysis

**Fig. 4:**
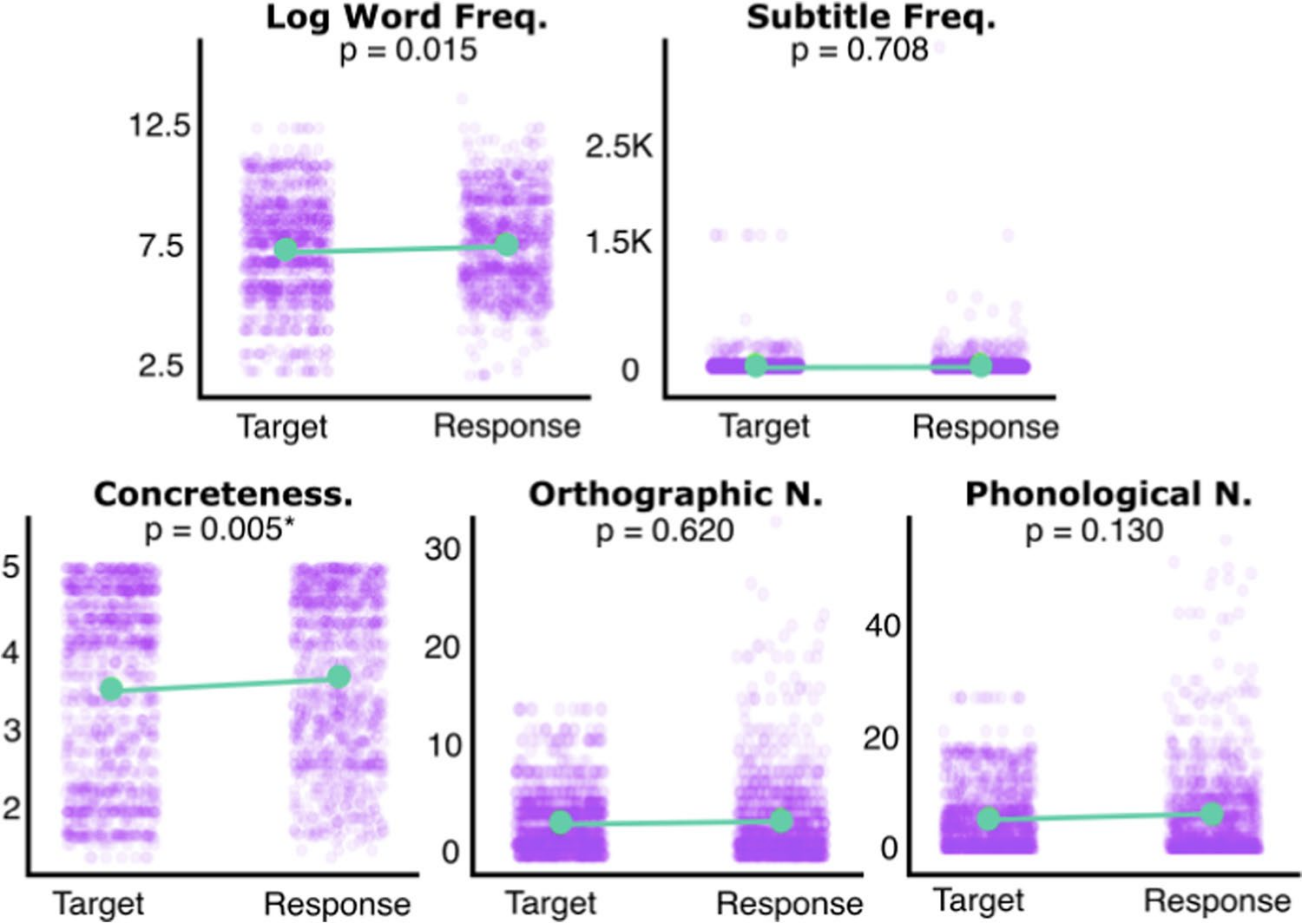
A visualisation of lexical characteristic differences between target words and paired lateralised reading error responses. Comparisons surviving correction for multiple comparisons (alpha level = 0.01) are starred. Purple points represent individual words whilst green represent group means. Green lines highlight difference in means between target and response words

First, a single-sample, two-tailed t-test was conducted to investigate whether response words were systematically different in length than target words in cases where patients committed lateralised reading errors. Response words were found to be significantly longer (mean length = 6.23 letters, SD = 1.05, range = 3–11) than target words (t(1710) = 9.1511, p < 0.001, 95% CI: 6.183–6.283). Finally, a binary logistic regression was conducted to identify lexical factors modulating the occurrence of neglect dyslexia by identifying characteristics which significantly predicted differences between target words and lateralised response errors. Overall, this model was found to be significant (F(1,2214) = 3.765, p = 0.002) with response words being significantly more concrete (estimate = 0.114, p = 0.005) than target words when neglect dyslexia reading errors were committed. All other considered lexical factors were found to be not significant (Fig. 4).

## Discussion

The purpose of this study was to determine whether word-centred neglect dyslexia can be simulated in healthy participants with normal reading abilities and, if so, to investigate the specific lexical and spatial parameters which modulate the occurrence of lateralised reading errors. Word-centred neglect dyslexia was successfully simulated, with a substantial proportion of reading errors committed in both horizontal and vertical conditions meeting the criteria for lateralised reading errors. The lateralisation of neglect dyslexia reading errors was found to be significantly predicted by cue lateralisation errors, with cue-congruent being more likely than cue-incongruent errors. Cue-congruent neglect dyslexia errors were more common in cases where spatial bias created by attentional cueing was congruent with innate spatial bias towards the initial letters of word stimuli. Cue-congruent errors were more common within vertical stimuli than in horizontal stimuli, and initial errors were slightly more common in horizontal orientation, regardless of cue location. Across all conditions, cue-congruent terminal errors occurred about three times as frequently as cue-congruent initial errors. Neglect dyslexia reading errors were not found to be shorter than target words (they were in fact significantly longer), but word concreteness was found to significantly predict differences between target and lateralised reading error words. Considered cumulatively, these findings provide novel insight into the mechanisms underlying word-centred neglect dyslexia.

This experiment was able to simulate word-centred neglect dyslexia reading errors in participants with normal reading abilities through an attentional cueing paradigm. Participants were found to commit > 50% lateralised reading errors when reading both horizontally and vertically presented words. This documented orientation insensitivity is characteristic of word-centred neglect dyslexia (Vallar et al., 2010). As in many clinical cases of word-centered neglect dyslexia, participants were found to commit comparatively more reading errors in vertical reading compared to horizontal reading (Moore & Demeyere, 2019, 2020, 2023). Additionally, the distribution of neglect dyslexia error types (predominantly substitution errors followed by omission and addition) agrees well with the error types documented in neglect dyslexia patients (Vallar et al., 2010). Specifically, previous work has found that the type of neglect dyslexia errors committed varies widely across patients, but substitution errors are the most common type of neglect dyslexia error, followed by omission and addition (Vallar et al., 2010). In this investigation, neglect dyslexia response errors were found to be an average of 0.23 letters longer than the relevant word stimulus. Again, this finding does not conflict with existing neglect dyslexia literature as word length encoding is generally spared in neglect dyslexia (Vallar et al., 2010) and many patients produce letter addition rather than omission errors (Vallar et al., 2010; Weinzierl et al., 2012). The neglect dyslexia errors simulated in this study primarily involved misreading stimuli as real words rather than non-words, which is also characteristic of neglect dyslexia (Moore & Demeyere, 2017; Vallar et al., 2010).

Notably, across all conditions, terminal (right-lateralised) neglect dyslexia errors were found to occur about three times as frequently as left-lateralised errors. This finding does not necessarily contradict with existing neglect dyslexia literature. While most of the documented cases of retinocentric and stimulus-centred neglect dyslexia have involved left-lateralised deficits (Vallar et al., 2010), most of the documented cases of word-centred neglect dyslexia have involved right-lateralised deficits (Moore & Demeyere, 2019, 2020, 2023). Importantly, the lateralised error pattern simulated in this experiment cannot be accounted for by ‘pseudoneglect’. This is because pseudoneglect produces subtle lateralised attentional biases in healthy adults (Jewell & McCourt, 2000), but could not be expected to produce the non-spatially lateralised (e.g., up/down) errors documented within this experiment’s vertical reading condition. This successful simulation of word-centred neglect dyslexia errors helps to provide insight into factors which may modulate the occurrence of this deficit in the absence of visuospatial neglect.

This finding is interesting when considered in the context of Hillis and Caramazza’s (1995) three-tier model of neglect dyslexia. First, Hillis and Caramazza (1995) do acknowledge that some apparent single dissociations between neglect dyslexia and neglect may arise due to differences in task attentional demands. However, Hillis and Caramazza’s (1995) model posits that word-centred neglect dyslexia is caused by a visuospatial attentional bias within the reference frame of internally coded, orientation canonical representations of words. According to this characterisation, word-centred neglect dyslexia impairment would not be expected to be simulated by introducing attentional cues in an externally defined reference frame. The cues employed in this investigation could be characterised as either retinocentric or stimulus-centred, but would not be expected to introduce attentional biases within internally coded spatial representations (Haywood & Coltheart, 2000; Hillis & Caramazza, 1995). If word-centred neglect dyslexia can be simulated in healthy participants using parameters that are not predicted to influence the occurrence of word-centred neglect dyslexia reading errors (such as the spatial cues used in this investigation), this suggests that parameters outside visuospatial neglect within an internally coded reference frame may be involved in word-centred neglect dyslexia. The results of this experiment provide preliminary insight into what some of these additional parameters may be.

The lateralisation of reading errors and the comparative impact of left versus right lateral cues documented in this investigation help elucidate factors underlying word-centred neglect dyslexia reading errors. Both left- and right-lateralised word-centred neglect dyslexia reading errors were found to occur regularly in this study, with cue-congruent (e.g., left neglect dyslexia error following a terminal cue) being more frequent than cue-incongruent errors. However, terminal reading errors were found to occur about three times as frequently as initial reading errors across all conditions. This difference in strength is likely due to the cue-induced leftward attentional bias combining with participant’s pre-existing leftward spatial attentional bias for reading stimuli (e.g. Gabrieli & Norton, 2012; Henderson, 1992; Scaltritti & Balota, 2013). Conversely, initial reading errors were likely less common due to participants’ leftward attentional reading bias competing with the right-ward cue-induced bias to produce a comparatively more balanced spatial attentional gradient (Gabrieli & Norton, 2012). Participants were found to be slightly more likely to commit initial errors in vertical reading conditions than horizontal reading conditions, regardless of cue location. This effect is likely due to the increased difficulty of the vertical condition and the corresponding increased error rate exhibited by participants (93% accuracy in horizontal reading vs. 80% in vertical conditions). Taken together, the comparative strengths of leftward versus rightward biases documented in this study demonstrate an interaction between inherent attentional biases in reading and the lateralised biases induced by attentional cues.

This imbalance between the occurrence of cue-congruent initial versus terminal errors also provides preliminary insight into how spatial attentional biases might modulate reading performance in healthy individuals. It is commonly asserted that healthy, single-word reading represents a parallel process in which both terminal and initial letters are processed simultaneously and matched to an existing lexical representation (Jackson & Coltheart, 2013; Scaltritti & Balota, 2013). These theories assert that serial reading (sequentially identifying graphemes from left-to-right) is expected to occur only in cases where readers do not have an existing lexical representation of the presented word (Jackson & Coltheart, 2013). These assertions do not clearly agree with the findings of this study. If healthy reading was a purely parallel process, it is unclear why biasing spatial attention towards one side of the stimulus would be expected to result in cue-congruent neglect dyslexia errors. In other words, if information about every letter in a word is encoded simultaneously, biasing attention to one side wouldn’t be expected to impact encoding of distal letters differently than more proximal ones. This issue is particularly apparent when considering that cue-congruent terminal errors were found to occur three times as often as cue-congruent initial errors in this study. These results agree well with previous literature demonstrating a processing advantage for letters in the first position of individual words, which occurs independently of lexical constraints (Scaltritti & Balota, 2013). Interestingly, this ‘first letter advantage’ has been documented to occur in both horizontally and vertically oriented word reading tasks (Scaltritti et al., 2018). Overall, the results of this study provide additional evidence that healthy, single-word reading may not be a purely parallel process. Instead, the results of this study suggest that spatial-attentional biases that operate independently of stimulus orientation may impact the efficiency (or order) in which graphemes are encoded.

Finally, the lexical analyses conducted in this investigation provide additional insight into the mechanisms underlying word-centred neglect dyslexia. In cases where participants committed neglect dyslexia reading errors, response words were found to contain significantly more letters than the target word. Patients with word-centred neglect dyslexia have been found to commit not only letter omission errors, but also letter substitutions and additions (Vallar et al., 2010). There has been some evidence that word length encoding is preserved in neglect dyslexia, helping to explain these diverse error patterns (Vallar et al., 2010). In cases where letters are not successfully encoded, patients may be able to rely on additional information sources (e.g., word length) to help infer the correct word (Moore & Demeyere, 2020). The lexical characteristic analyses conducted in this study provide a preliminary indication that other sources of information may be relied upon when patients are committing neglect dyslexia errors. Mainly, in cases where participants made lateralised reading errors, response words were found to have a significantly higher concreteness rating than target words. Previous studies have suggested that more concrete words are more available during recall tests (Brysbaert et al., 2014; Miller & Roodenrys, 2009). This implies that in cases where participants are attempting to infer or ‘fill in’ correct responses in cases where not all letters have been encoded, participants could potentially be responding based on which word they can most quickly call to mind (Moore et al., 2020). It therefore seems plausible that a similar inferential mechanism might underly reading error patterns in patients with word-centred neglect dyslexia. However, additional research is needed before these preliminary implications can be interpreted with confidence and to eliminate any other possible explanations for the errors observed in word-centred neglect dyslexia.

As with any neuropsychological syndrome, understanding the specific cognitive mechanisms underlying word-centred neglect dyslexia is a critical precursor to designing effective and targeted rehabilitation strategies for patients living with this reading impairment. The present study adds to the existing literature by providing additional evidence that spatial-attentional deficits might not be the only mechanism contributing to the occurrence of word-centred neglect dyslexia. Specifically, these findings suggest that word-centred neglect dyslexia may involve complex inferential and inhibitory interactions as participants attempt to predict correct target words from incomplete letter encoding (Moore & Demeyere, 2019, 2020). Future research can aim to determine whether similar effects can be documented in a substantive clinical sample of patients exhibiting word-centred neglect dyslexia.

### Limitations

This study employs data from participants with normal reading abilities to gain insight into the factors that may be modulating word-centred neglect dyslexia in the patient population. While the presented findings suggest that an alternate model of word-centred neglect dyslexia is needed, additional work is needed to thoroughly test and evaluate potential alternate models (such as Moore & Demeyere’s (2023) proposed cognitive inhibition model) before any potential alternate model can be confidently accepted. This study aimed to provide insight into what word-centred neglect dyslexia is not, and additional data are needed before confident conclusions about what specific cognitive mechanisms may underly word-centred neglect dyslexia can be drawn.

It is not entirely clear whether any healthy participant simulation can serve as an adequate proxy of a neuropsychological syndrome. This study simulates reading impairments in a dual-task design that may not be entirely analogous to naturalistic reading, which is rarely a secondary task (Behrmann et al., 1991; Jackson & Coltheart, 2013). It is also possible that the error pattern simulated in this study may represent participants ‘filling in’ information that was not encoded rather than exhibiting the biases in employing encoded information which are seen in word-centred neglect dyslexia (Moore & Demeyere, 2020, 2023). However, the behavioural patterns identified in this study agree closely with those documented within the word-centred neglect dyslexia population. In addition to this, past work has suggested that reading performance in patients with neglect dyslexia is modulated by attentional cues in a similar manor to the effects documented in the present study (Behrmann et al., 1990; Cubelli & Beschin, 2005; Riddoch et al., 1990). These findings indicate that initial evidence can be provided by such simulation paradigms. However, stronger evidence in support of the implications of this study can be provided by future studies employing similar cueing paradigms in clinical cases of neglect dyslexia.

Additionally, each word stimulus was repeated multiple times throughout the study. This approach was chosen in order to minimise systematic differences due to the specific word stimuli used in each condition. However, this practice may have led to some degree of learning effect. Given that participants were found to commit a range of lateralised and unlateralised reading errors across all conditions and that blocks were randomised between participants, it seems unlikely that these potential learning effects had any significant impact on the results of this study. If systematic learning effects were present, reading performance would be expected to improve within later blocks relative to earlier blocks. However, performance accuracy was not different across different blocks when blocks were coded according to order of completion, indicating the absence of any systematic learning effects.

Further, it is challenging to objectively define values associated with lexical characteristics, especially in experiments involving multiple participants. This investigation employed standard lexical characteristic values (Balota et al., 2007), but it is not clearly possible to determine whether these are consistent and representative across participants. For example, one participant may have more familiarity with one given word due to exposure, culture or background regardless of that word’s standardised familiarity value. This natural variation likely introduced some degree of noise into this study’s lexical characteristic analyses. A large number of trials, large sample group, and stringently corrected statistical comparisons have been employed in order to control for this potential source of variance.

Finally, word-centred neglect dyslexia is a highly heterogenous condition and large, representative and standardised group studies are needed before these findings can be effectively generalised to the patient population. The findings presented in this study provide initial insight into some of the mechanisms that may be modulating the occurrence of lateralised reading errors in neglect dyslexia that can be explored in future larger-scale investigations of the disorder.

## Conclusions

The findings of this study demonstrate that word-centred neglect dyslexia-like reading errors can be simulated using attentional cues in a sample of healthy readers. Left-lateral cues were found to elicit more reading errors than right lateral cues, illustrating the interaction between existing spatial attentional biases in reading and cue-induced biases. In cases where participants did make a neglect dyslexia error, they tended to produce a response that had a significantly higher concreteness rating than the target word. Overall, these findings suggest that at least some cases of word-centred neglect dyslexia may not be fully accounted for by the neglect syndrome, but may also involve complex interactions from lexical, inhibitory and non-pathological spatial bias mechanisms.

### Supplementary Information

Below is the link to the electronic supplementary material.

Supplementary file1 (DOCX 35 KB)

## Data Availability

The datasets generated during and/or analysed during the current study are available in the Open Science Framework repository, https://osf.io/nkzb8/.

## Code availability

All experimental and analysis code associated with this project is available on the Open Science Framework, https://osf.io/nkzb8/.
