# Supplementary material for: Attentional and lexical factors underlying word-centred neglect dyslexia errors in healthy readers
Source: Atten Percept Psychophys. 2023 Jul 6;86(1):312–25. doi: 10.3758/s13414-023-02753-x (PMC10769981; doi:10.3758/s13414-023-02753-x)
Supplement: Supplementary file 1 — Supplementary file1 (DOCX 35 KB) [file 13414_2023_2753_MOESM1_ESM.docx]

**Attentional and lexical factors underlying word-centred neglect dyslexia errors in healthy readers**

James Gurd^1^, Nele Demeyere^1^, & Margaret Jane Moore^1,2^

^1^Department of Experimental Psychology, University of Oxford, Oxford, UK

^2^Queensland Brain Institute, University of Queensland, Brisbane, UK

**Appendix 1**

| abused | brains | errand | kitten | parrot | sonnet |
| --- | --- | --- | --- | --- | --- |
| acuter | branch | fabled | laptop | pastry | sparks |
| agreed | brandy | failed | latest | payday | stable |
| airbag | budget | fellow | lawful | peanut | staged |
| airbed | bulbed | forest | layout | person | stinky |
| airway | bullet | forget | legend | piglet | stripe |
| alarms | button | format | linear | planet | summit |
| allied | cannot | fouled | linked | plants | sunbed |
| allies | caring | frozen | listen | poetry | sunlit |
| anklet | carpet | golden | manage | potter | sunset |
| anyone | carrot | graced | manger | pushed | surely |
| anyway | carton | graven | margin | raided | swiped |
| appeal | catkin | hatred | marshy | ranger | talent |
| appear | catnip | hearth | martin | really | target |
| armpit | center | hearts | mayday | ripsaw | teabag |
| attics | closer | hidden | mayhem | robust | teapot |
| ballet | closet | hobbit | menace | rodent | tenant |
| ballot | clover | holder | mobile | roofed | tiffin |
| bangle | copper | honest | monkey | rowing | tiptoe |
| barely | cotton | hornet | napkin | runway | toilet |
| barest | coward | hotbed | offcut | scales | toward |
| barman | cowboy | hotdog | office | scared | turnip |
| barrow | crafts | hotpot | offset | scores | wallet |
| begins | cutter | impact | orally | search | warden |
| bellow | damage | impart | outfit | season | washed |
| bestow | dearly | inkpad | outfox | seesaw | willow |
| betray | dimwit | itself | output | shared | wither |
| blocks | dinner | keypad | outrun | slater | within |
| blower | driver | kidnap | palace | sliced | wooden |
| bowtie | ending | killed | parent | slowed | yellow |
